# Multivariate regression analysis of structural MRI connectivity matrices in Alzheimer’s disease

**DOI:** 10.1371/journal.pone.0187281

**Published:** 2017-11-14

**Authors:** Javier Rasero, Nicola Amoroso, Marianna La Rocca, Sabina Tangaro, Roberto Bellotti, Sebastiano Stramaglia, for the Alzheimer’s Disease Neuroimaging Initiative

**Affiliations:** 1 Biocruces Health Research Institute. Hospital Universitario de Cruces. E-48903, Barakaldo, Spain; 2 Dipartimento di Fisica, Universitá degli Studi Aldo Moro, Via Orabona,4, 70126 Bari, Italy; 3 INFN, Sezione di Bari, via Orabona 4, 70126 Bari, Italy; 4 TIRES-Center of Innovative Technologies for Signal Detection and Processing, Università degli Studi Aldo Moro, Bari, Italy; Banner Alzheimer’s Institute, UNITED STATES

## Abstract

Alzheimer’s disease (AD) is the most common form of dementia among older people and increasing longevity ensures its prevalence will rise even further. Whether AD originates by disconnecting a localized brain area and propagates to the rest of the brain across disease-severity progression is a question with an unknown answer. An important related challenge is to predict whether a given subject, with a mild cognitive impairment (MCI), will convert or not to AD. Here, our aim is to characterize the structural connectivity pattern of MCI and AD subjects using the multivariate distance matrix regression (MDMR) analysis, and to compare it to those of healthy subjects. MDMR is a technique developed in genomics that has been recently applied to functional brain network data, and here applied to identify brain nodes with different connectivity patterns, in controls and patients, because of brain atrophy. We address this issue at the macroscale by looking to differences in individual structural MRI brain networks, obtained from MR images according to a recently proposed definition of connectivity which measures the image similarity between patches at different locations in the brain. In particular, using data from ADNI, we selected four groups of subjects (all of them matched by age and sex): HC (healthy control participants), ncMCI (mild cognitive impairment not converting to AD), cMCI (mild cognitive impairment converting to AD) and AD. Next, we built structural MRI brain networks and performed group comparison for all the pairs of groups. Our results were three-fold: (i) considering the comparison of HC with the three other groups, the number of significant brain regions was 4 for ncMCI, 290 for cMCI and 74 for AD, out of a total of 549 regions; hence, in terms of the structural MRI connectivity here adopted, cMCI subjects have the maximal altered pattern w.r.t. healthy conditions. (ii) Eight and seven nodes were significant for the comparisons AD-ncMCI and AD-cMCI, respectively; six nodes, among them, were significant in both comparisons and these nodes form a connected brain region (corresponding to hippocampus, amygdala, Parahippocampal Gyrus, Planum Polare, Frontal Orbital Cortex, Temporal Pole and subcallosal cortex) showing reduced strength of connectivity in the MCI stages; (iii) The connectivity maps of cMCI and ncMCI subjects significantly differ from the connectome of healthy subjects in three regions all corresponding to Frontal Orbital Cortex.

## Introduction

Alzheimer’s disease (AD) is a progressive, neurodegenerative disease accounting for most cases of dementia after the age of 65: the prevalence of clinically manifested AD is about 2% at the age of 65 years, it increases to about 30% at the age of 85 years [1]. AD is characterized by an accumulation of beta-amyloid plaques and neurofibrillary tangles composed of tau amyloid fibrils [2] associated with synapse loss and neurodegeneration leading to long-term memory impairment and other cognitive problems. There is currently no known treatment that slows down the progression of this disorder. It is now accepted that the neurodegenerative cascade in AD begins in the brain years, decades even, before the clinical and radiological manifestations of the illness. The dementia is preceded by a prodromal phase of mild cognitive impairment, and this, in turn, by a pre-clinical phase of variable duration. The notion of MCI, a disorder situated in the spectrum between normal age-related cognitive decline and dementia, has varied over the past 2 decades. MCI has been classified into different broad categories depending on memory performance and the number of impaired cognitive functions [3]. An accurate prediction of conversion from MCI to AD can help clinicians to evaluate AD risk pre-symptomatically, initiate treatments at early stage, and monitor their effectiveness [4, 5]. However, the group of MCI is very heterogeneous, and not all MCI patients convert to AD [6]. The annual rate in which MCI progresses to dementia varies between 8% and 15% per year [7]. The amnestic subtype of MCI is more prevalent than non-amnestic one [8], and it has a significantly higher annual conversion rate to AD, between 30% [9, 10] to 40% [11].

Many neuroimaging studies addressed the conversion from MCI to AD (see [12] and references therein). In particular, it was shown that the hippocampus volume and the volume from other subcortical structures at MCI were well correlated to a worse progression to AD, with accuracy of about 65% in the prediction from MCI to AD [13]. Particularly important is the choice of the hippocampal segmentation protocol [14]. Rather than assuming that specific brain regions are going to be affected by AD, other authors achieved a better accuracy in the prediction from MCI to AD (achieving values of about 80% accuracy) by performing a blind approach including multiple regions of interest [15–17]. Despite extensive research shedding light into this problem, the precise mechanisms and clinical variables responsible for the progression from MCI to AD have not been fully characterized, mainly due to the lack of time-resolved longitudinal studies in large populations.

Human brain networks [18, 19] can be constructed using multimodal neuroimaging techniques in vivo. Nowadays, functional MRI (fMRI) and diffusion tensor imaging (DTI) are the two main modalities to build individual brain networks [20, 21]. Besides fMRI and DTI, structural MRI has recently attracted increasing attention in delineating whole-brain morphological connectivity patterns by calculating interregional morphological correlations across a cohort of participants [22, 23]. Compared with fMRI and DTI, MRI has an easy access, high signal-to-noise ratio, and relative insensitivity to artifacts (e.g., head motion). MRI-based brain networks are thus a promising approach to characterize brain organization under both healthy and pathological conditions [24]. Nevertheless, it should be noted that the methodology of [22, 23] can obtain only one network for a group of participants, while ignoring inter-individual variability. This makes problematic the examination of brain-behavior relationships and health-disease classification. Methods for the construction of a network from an individual structural MR image have been reported [25–27], but not yet widely applied. In [28] the topology of these networks in healthy and AD patients was analyzed, whilst in [29] the graph properties and their connection with cognitive impairment in early- and late-onset Alzheimer’s disease was studied; in [30] the brain properties in schizophrenia were studied.

Regarding modelling and description of the brain, a number of different graph theory strategies have been widely used in recent years [31–33]. Two approaches are mainly adopted [34]: (i) voxel-wise and (ii) region of interest analyses. We adopt here a patch-based approach [27] which combines the ease of interpretation typical of voxel-wise descriptions (while avoiding its huge computational burden [35]) with the robustness of region of interest (ROI) strategies, without the intrinsic segmentation errors affecting ROI approaches [36]. Brain diseases have diffuse effects, affecting multiple voxels but not necessarily corresponding to entire anatomical structures, nonetheless whole brain investigations cannot have the required sensitivity, especially looking for subtle effects on millions of voxels with typically small size data samples. Therefore, here we construct individual MRI brain networks from images provided by the Alzheimer’s Disease Neuroimaging Initiative (ADNI), and corresponding to healthy controls, MCI subject converting or not to AD, and AD patients; we look for brain regions whose map of connectivity, with the rest of the brain, is significantly altered due to the disease. To this aim we use the method in [37] where techniques from genome wide association studies, coping with the problem of huge number of comparisons, were applied to functional connectome data.

## Materials and methods

### Materials

The MR images used in this paper were obtained from ADNI database http://adni.loni.usc.edu [38]. ADNI was launched in 2003 by the Nat. Inst. on Aging (NIA), the Nat. Inst. Biomedical Imaging and Bioengineering (NIBIB), the Food and Drug Administration (FDA), private pharmaceutical companies and non-profit organizations, as a $60 million, 5-year public-private partnership. ADNI’s main goal has been to test whether serial magnetic resonance imaging (MRI), positron emission tomography (PET), other biological markers, and clinical and neuropsychological assessment can be combined to measure the progression of mild cognitive impairment (MCI) and early Alzheimer’s disease (AD). Determination of sensitive and specific markers of very early AD progression is intended to aid researchers and clinicians to develop new treatments and monitor their effectiveness, as well as to lessen the time and cost of clinical trials. The Principal Investigator of this initiative is Michael W. Weiner, MD, VA Medical Center and Univ. California—San Francisco. ADNI subjects have been recruited from over 50 sites across the U.S. and Canada. Currently, around 1500 adults were recruited in the different ADNI initiatives, ages 55 to 90. The follow up duration of each group is specified in the protocols for ADNI-1, ADNI-2 and ADNI-GO, see further information in www.adni-info.org.

A total number of N = 316 subjects were used in this study; MR images were selected and downloaded from ADNI database, belonging to 4 different groups: healthy controls HC (*N*_1_ = 80), subjects with mild cognitive impairment not converting to Alzheimer Disease ncMCI (*N*_2_ = 82), subjects with mild cognitive impairment converting to Alzheimer Disease cMCI (*N*_3_ = 70) and subjects affected by the disease AD (*N*_4_ = 84). Some subjects belong to a benchmark dataset selected in order to obtain a compact yet representative sample of ADNI [39]. Remaining subjects were randomly sampled from the whole ADNI in order to match the demographic characteristics of benchmark subjects, making sure that each subject is not considered twice. Age and sex were balanced across groups (see Tables 1, 2 and 3) respectively, using a t-test and chi-squared test. cMCI subjects had converted to AD in a time range of (30,108) months subsequent to the initial assessment. All 316 participants underwent whole-brain MRI at 34 different sites. Both 1,5 T and 3,0 T scans were included. ADNI images consisted of MPRAGE MRI brain scans, which were normalized with the MNI152 brain template of size of 197 × 233 × 189 mm^3^ and resolution of 1 × 1 × 1 mm^3^. Accordingly, from now onwards mm^3^ and voxels are interchangeably used.

**Table 1:** Demographic groups information.

Group Label	Age	Sex (M/F)
HC	74.95 (60-89)	(43/37)
ncMCI	75.48 (60-89)	(52/30)
cMCI	74.73 (55-87)	(39/31)
AD	75.95 (60-90)	(45/39)

**Table 2:** P-values group matrices from two sample t-test for age showing that groups are appropriately balanced.

	HC	ncMCI	cMCI	AD
HC	-	0.598	0.843	0.537
ncMCI	0.598	-	0.525	0.914
cMCI	0.843	0.525	-	0.476
AD	0.537	0.914	0.476	-

**Table 3:** P-values group matrices from *χ*^2^ test for sex showing that groups are appropriately balanced.

	HC	ncMCI	cMCI	AD
HC	-	0.276	0.939	1
ncMCI	0.276	-	0.424	0.259
cMCI	0.939	0.424	-	0.918
AD	1	0.259	0.918	-

### Image processing

Image processing was carried out with the Oxford FMRIB library FSL [40]. Firstly, MRI scan intensity differences, yielded by bias field, were normalized, then intra-cranial regions were extracted with the FSL Brain Extraction Tool (BET). After intensity normalization and brain extraction, a spatial normalization was performed to co-register the different images into a common coordinate space. The MNI152 was adopted as reference template. An affine registration was performed with the FSL Linear Registration Tool (FLIRT) with a standard parameter configuration. The method adopted here for network modeling is based on the idea that anatomical regions should roughly overlap in order to be robust to subtle local differences, due for example to subject morphological variability, or small registration failures. Once the MRI scans and the template had been co-registered they shared the same reference space and dimensions. Thus, using the template brain coordinates, we automatically divided the brain of each subject into the two hemispheres by the medial longitudinal fissure. Starting from this sagittal plane, it was possible to uniformely cover each hemisphere with an equal number of rectangular *ℓ*_1_ × *ℓ*_2_ × *ℓ*_3_ boxes, from now onward referred to as patches, covering the whole brain. Patches provide a gray matter volume measure and its spatial distribution and are not chosen to represent predefined anatomical areas, in contrast to region of interest approaches. We adopted a patch size of 3000 voxels, corresponding to the optimal dimensions 10 × 15 × 20 mm^3^. Thus, 549 patches were used to cover the whole brain, as explained in the next section. The patches were considered nodes of a network whose connections represent the degree of similarity between them. To obtain an anatomical interpretation of brain nodes, it is important to relate them to anatomical areas of interest for the disease. Nodes, identified as significant by the proposed approach, were localised on the reference template and the corresponding atlas. We adopted Harvard-Oxford cortical and sub-cortical structural atlases [41].

### Definition of connectivity between patches

For each subject and for each pair of patches, the corresponding measure of connectivity was evaluated as follows. The intensity values of voxels within a patch were reshaped to form a vector of length *ℓ*_1_ *ℓ*_2_ *ℓ*_3_, in the same way for the two patches; then the Pearson’s correlation coefficient between these two vectors was evaluated and taken as the connectivity strength between the two nodes associated to the patches. Pearson’s correlation was chosen in [27] to model the effects of atrophy, as it is fast to implement and compute, simple to understand and interpret, and it does not require any scaling or centering of the patches, being intrinsically normalized. In addition, Pearson correlation is a similarity criterion that associates corresponding voxels within patches, therefore taking into account spatial relationships between voxels, differently from [26] where the Kullback divergence, between the intensity histograms of the intensity in pairs of ROIs, was considered. The Pearson correlation between small cubes (consisting of 27 voxels) has been already used in [25] to construct individual MRI networks, obtaining networks of about 7000 nodes. In order to provide a robust description of the brain, in [27] it has been proposed to consider patches of about 3000 voxels, i.e. on a dimensional scale far higher than the voxel, but not as large as in a ROI description.

In order to fix the optimal dimension for the brain subdivision in rectangular patches, we explored different patch sizes and for each size we computed some intensity related features (degree, strength, inverse participation, and others) to assess the information content provided by those patches; we fed a random forest classifiers with these features in a 5-fold cross-validation framework and evaluated the classification accuracy for discriminating healthy control and AD patients. Experimental results showed an accurate and stable classification performance within the [2250;3200] voxel (mm^3^) range, corresponding approximately to 500 patches, with variations lower than 5%. The best accuracy (≈ 88%) was obtained with *N* = 549 patches. Accordingly, we have adopted this patch size, which leads for each subject *u* to a 549 × 549 structural MRI network $C_{u}={\left\{c_{u}^{ij}\right\}}_{i,j=1,\ldots ,N}$

### Multivariate distance matrix regression analysis

A cross-group analysis has been performed using the Multivariate Distance Matrix Regression (MDMR) approach proposed in [37], which allows testing the variation of distance in connectivity patterns between groups as a response of the Alzheimer’s progression. As in [37], for each fixed brain node i, and for each pair of subjects (u,v), the vector representing the connectivity pattern of i with all the brain is considered, for both subjects u and v, ${\left\{c_{u}^{ij}\right\}}_{j=1,\ldots ,N}$ and ${\left\{c_{v}^{ij}\right\}}_{j=1,\ldots ,N}$. Then a distance between u and v is defined as:

$$\begin{array}{c} d_{uv}^{i}=\sqrt{2(1-r_{uv}^{i})} \end{array}$$ (1)

where $r_{uv}^{i}$ is the Pearson correlation between connectivity patterns of i for subjects u and v, ${\left\{c_{u}^{ij}\right\}}_{j=1,\ldots ,N}$ and ${\left\{c_{v}^{ij}\right\}}_{j=1,\ldots ,N}$. Thus, the collection of distances amounts, at fixed brain region i, to a distance matrix in the subject space. Note that both u and v vary in the two groups, hence the distance matrix contains both intra-group distances and inter-group ones.

Next, we applied MDMR to perform cross-group analysis as implemented in R [42]. MDMR yielded a pseudo-F estimator (analogous to that F-estimator in standard ANOVA analysis), which addresses significance of the between-group variation as compared to within-group variations, details can be found in [37]. When two groups, of cardinality *n*_1_ and *n*_2_ respectively, are being compared and the regressor variable is thus categorical, the approach of [37] is equivalent to what follows. One firstly calculate the total sum of squares as:

$$\begin{array}{c} SS_{T}=\frac{1}{n}\sum_{u=1}^{N}\sum_{v=u+1}^{N}d_{uv}^{2} \end{array}$$ (2)

with *n* = *n*_1_ + *n*_2_ being the total number of subjects. In this way, one gets a different *SS*_*T*_ for each region i. Similarly, the within-group sum of squares can be written as

$$\begin{array}{c} SS_{W}=\frac{1}{n_{1}}\sum_{u=1}^{n}\sum_{v=u+1}^{n}d_{uv}^{2}\epsilon_{uv}^{a}+\frac{1}{n_{2}}\sum_{u=1}^{n}\sum_{v=u+1}^{n}d_{uv}^{2}\epsilon_{uv}^{b} \end{array}$$ (3)

where $\epsilon_{uv}^{a}$ is one if *u* and *v* belong to the first group and zero otherwise; similarly $\epsilon_{uv}^{b}$ is one if *u* and *v* belong to the second group and zero otherwise. The between-group variation is then given by *SS*_*A*_ = *SS*_*T*_ − *SS*_*W*_, and the pseudo-F statistic of [37] amounts to the following:

$$\begin{array}{c} F=\left(N-1\right)\frac{SS_{A}}{SS_{W}} \end{array}$$ (4)

As it was acknowledged in [37], the pseudo-F statistic is not distributed like the usual Fisher’s F-distribution under the null hypothesis. Accordingly, we randomly shuffled the subject indices and computed the pseudo-F statistic each time. A p-value is computed by counting those pseudo F-statistic values from permuted data greater than that from the original data, and divide by the total number of performed permutations. Finally, we controlled for type I errors due to the independent statistical performed tests by false discovery rate corrections. We required 1% significance so as to discuss more accurate findings.

## Results

### Comparisons with healthy subjects

The comparison between HC and each of the other three groups aims at identifying brain regions whose connectivity map is altered w.r.t. healthy conditions. Application of MDMR, at 1% significance after false discovery rate correction, leads to the number of brain regions, with significant different patterns in the two groups, depicted in Table 4.

**Table 4:** Number of altered regions, in the MCI and AD groups, w.r.t. HC.

ncMCI	cMCI	AD
4	290	74

**Fig 1:**
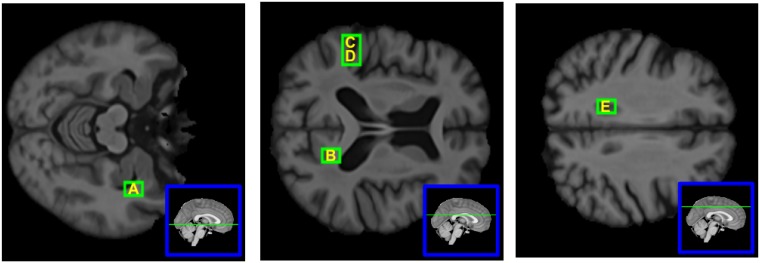
Brain nodes which are altered only in AD. The five brain nodes which are altered only in AD. Their patches correspond to the following anatomical structures: (A) right hemisphere: Hippocampus, Amygdala, Temporal Fusiform Cortex (posterior division), Planum Polare; (B) right hemisphere: Cingulate Gyrus, Lateral Ventricle, Precuneous Cortex; (C) left hemisphere: Superior Temporal Gyrus, Postcentral Gyrus; (D)left hemisphere: Planum Temporale, Central Opercular Cortex; (E) left hemisphere: Cingulate Gyrus, Lateral Ventricle.

The four regions altered in ncMCI are also found altered in cMCI but not in AD. Out of the 74 regions altered in AD, 69 were already found altered in cMCI whilst five regions were altered only for AD and not for MCI groups: the alteration of the patterns of these five regions, hence, is peculiar of the AD disease. The location of these five patches, in the brain, is depicted in Fig 1. We report that at 5% significance the number of altered regions would have been 99 (ncMCI) 421 (cMCI) and 323 (AD).

### Comparing MCI groups with AD

At 1% significance after false discovery rate correction, comparing ncMCI and AD, the nodes that are found to have significantly different pattern are eight, whilst comparing cMCI and AD the significant nodes are seven. All these regions are not significant in the comparison HC-AD.

**Fig 2:**
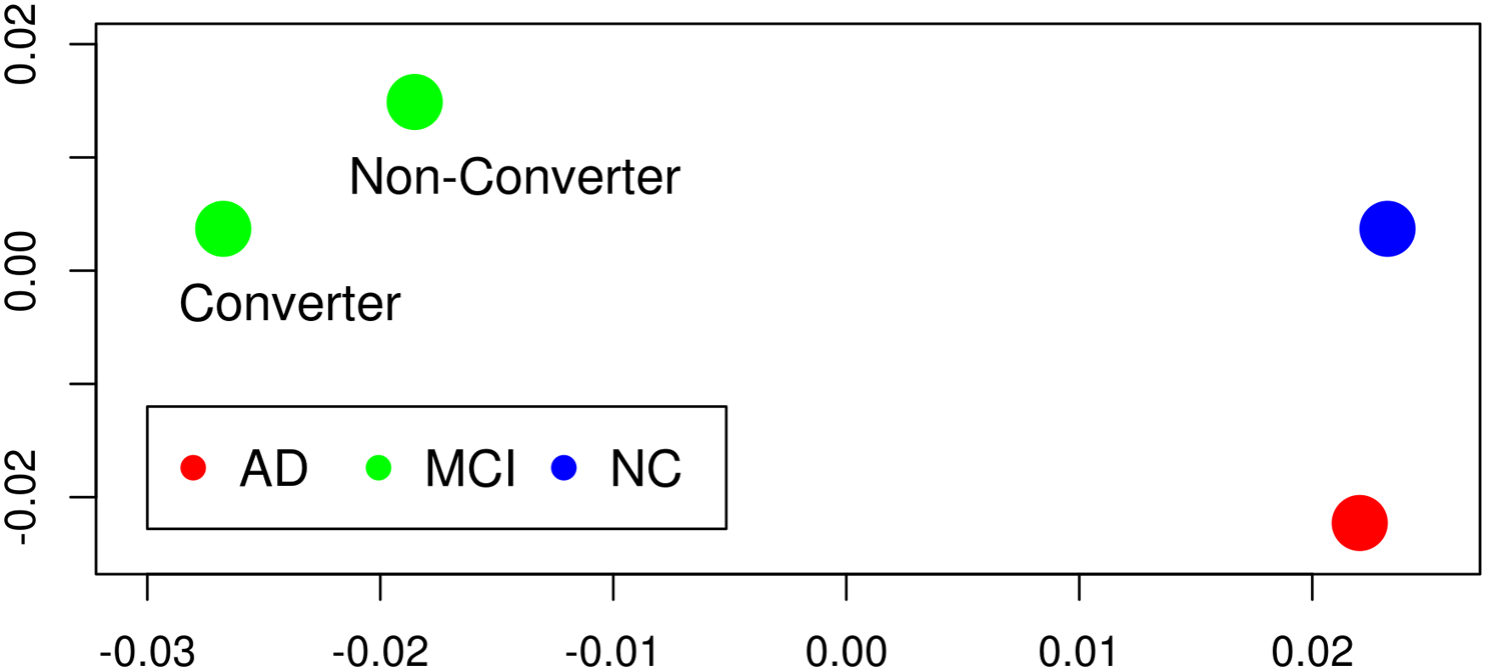
Multidimensional scaling from signficant MCI-AD nodes. In order to visualize the peculiar pattern of the region corresponding to the six nodes that are significant when AD patients are compared with MCI, the four groups are represented here, using multidimensional scaling applied to the average connectivity map of these brain regions, as explained in the text. The plot clearly shows that, in AD, the connectivity pattern of this region is similar to those corresponding to healthy conditions. As we are dealing with distances between arrays of Pearson correlation coefficients, the units of both axis are dimensionless.

**Fig 3:**
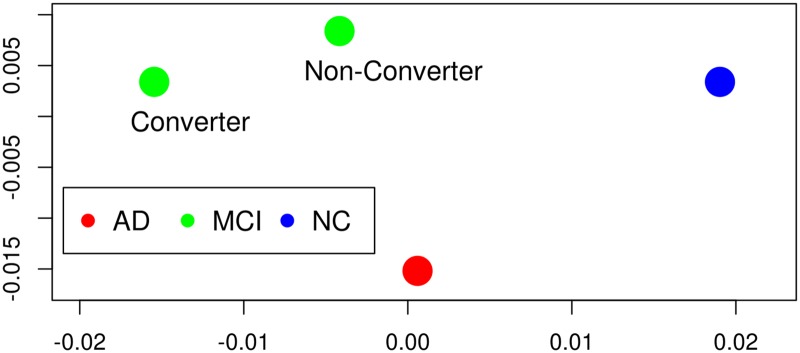
Multidimensional scaling from all brain regions. The same as the previous figure but all the brain regions have been used to provide the input to multidimensional scaling. It shows that also globally in AD the connectivity is closer to those of controls than MCI states, although to a lesser extent than the six regions of Fig 1. As we are dealing with distances between arrays of Pearson correlation coefficients, the units of both axis are dimensionless.

Six regions, out of these, are significant for both comparisons ncMCI-AD and cMCI-AD; they include also the four significant regions for the comparisons HC-ncMCI and HC-cMCI. In order to visualize the peculiar pattern of these six regions, within each group we averaged their connectivity pattern with the whole brain, obtaining a 6 × 549 matrix for each group. Then, a mutual distance between these matrices have been evaluated as the mean of the absolute values of the differences of entries. Using multidimensional scaling [43] we obtained the four points, in two dimensions, whose Euclidean distances are closest to the distances among submatrices, as depicted in Fig 2; for comparison, in Fig 3 we also depict the four points that we obtain applying the same procedure to the whole 549 × 549 matrices. These six patches are thus characterized by a pattern which is peculiar to the MCI conditions.

**Fig 4:**
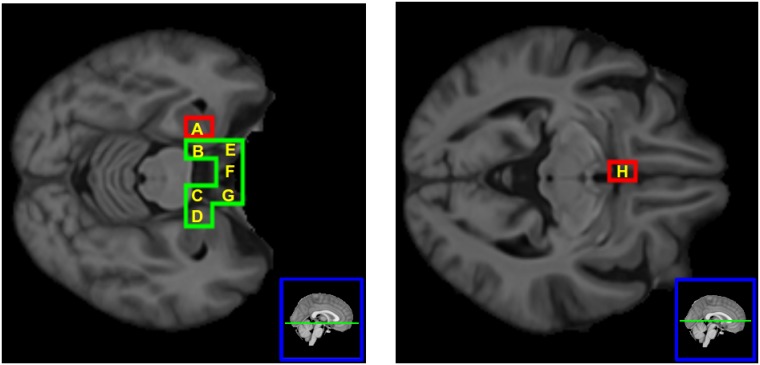
The brain nodes significantly differing in MCI and AD. Their patches correspond to the following anatomical structures: (A) left hemisphere: Hippocampus, Amygdala, Planum Polare; (B) left hemisphere: Parahippocampal Gyrus; (C) right hemisphere: Parahippocampal Gyrus; (D) right hemisphere: Hippocampus, Amygdala, Planum Polare; (E) left hemisphere: Frontal Orbital Cortex, Temporal Pole; (F) Inter-hemispheric: Subcallosal Cortex. (G) right hemisphere: Frontal Orbital Cortex, Temporal Pole; (H) left hemisphere: Subcallosal Cortex. Green patches are significant for both ncMCI and cMCI, whilst red patches are significant only for ncMCI.

It is also worth stressing that two brain regions are significant only for ncMCI, i.e. they are relevant for the not converting MCI condition. In Fig 4 we depict the brain nodes highlighted by the comparison between MCI and AD.

We report that at 5% significance the number of significant regions in the comparison with AD would have been 22 (ncMCI) and 17 (cMCI).

### Comparing ncMCI and cMCI

**Fig 5:**
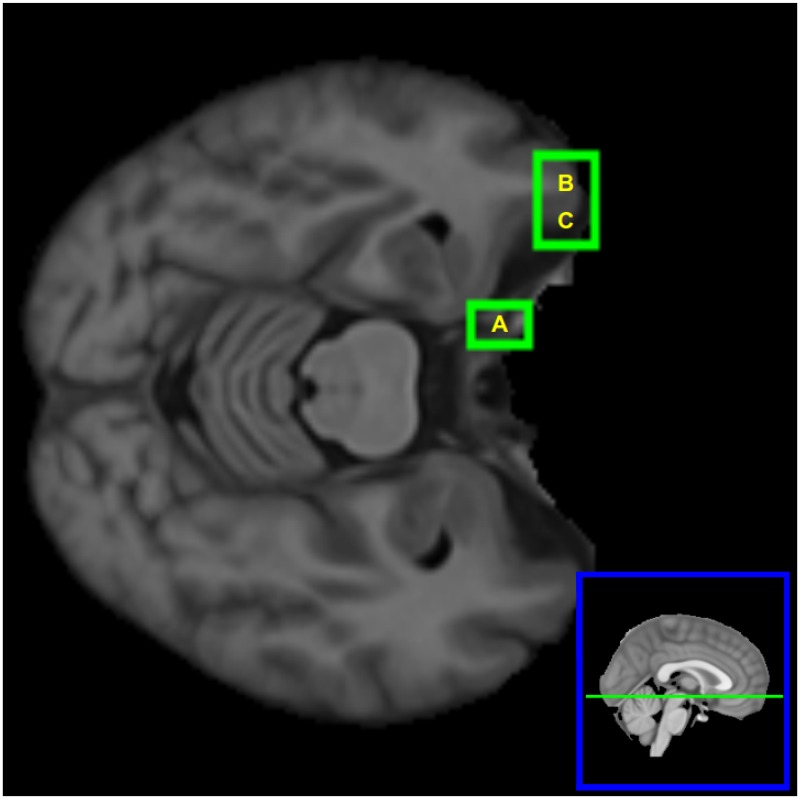
The three significant regions for the comparison ncMCI-cMCI. Their patches are all in the left hemisphere and correspond to the following anatomical structures: (A) Frontal Orbital Cortex, Temporal Pole; (B) Frontal Pole, Frontal Orbital Cortex; (C) Frontal Pole, Frontal Orbital Cortex.

The application of MDMR to the two groups ncMCI and cMCI did not identify any significant region, although the cMCI shows an huge number of altered brain regions w.r.t. HC, differently from ncMCI which shows just four. Such a big difference, in the two comparisons ncMCI-HC and cMCI-HC, suggested us to project the connectivity maps of MCI subjects onto the average connectome of healthy subjects, as follows. Consider the brain region *i*, and let *h* be the connectivity vector of i averaged over the set of HC subjects. For every MCI subject, *u*, we denote *c*_*i*_(*u*) the Pearson correlation between *h* and the connectivity vector of region i in subject *u*. The farthest *c*_*i*_(*u*) from one, the more altered from healthy conditions the pattern of connectivity of brain region i in subject u. For each node i, we perform a nonparametric test (Wilcoxon ranksum) against the hypothesis that the 82 values of this quantity for the ncMCI group come from a distribution with median higher than those of the cMCI group, and use the Bonferroni correction for the 549 multiple comparison. Three regions are found to be statistically significant at 1% significance, and they are depicted in Fig 5.

### Comparison of the average strength in the groups

We note that the Pearson correlation between two patches depends on the shape of the connectivity patterns but not on their amplitude. Hence the MDMR analysis can be complemented by an analysis of the strength of connectivity that we calculate as the mean of the absolute value over all entries of the structural MRI network, for each subject. We perform a nonparametric test (Wilcoxon ranksum) against the hypothesis that the 80 values of this quantity for the HC group come from a distribution with median higher than those of the three other groups, obtaining the p-values depicted in Table 5. These values show that the strengths are significantly higher in controls than MCI, whilst statistically it is not possible to differentiate controls and AD on the basis of the average strength of networks. Moreover, cMCI is the group with smallest p-value, i.e. the most altered w.r.t. healthy conditions.

**Table 5:** P-value of the comparison of the mean strength of connectivity, i.e. the mean of the absolute value of all entries of the structural MRI network of healthy subjects, with the other groups. cMCI is the group with smallest p-value, i.e. the most altered w.r.t. healthy conditions.

ncMCI	cMCI	AD
3.5 × 10^−4^	7.1 × 10^−6^	0.57

### Non-parametric distance between connectivity patterns

In the standard application of MDMR, the distance between connectivity patterns is defined in terms of Pearson correlation. To assess the robustness of our results w.r.t. of the choice of the distance, we have repeated our analysis using a non-parametric measure of the distance which reads as follows

$$\begin{array}{c} d_{uv}^{i}=\sqrt{2(1-\rho_{uv}^{i})}\;, \end{array}$$ (5)

where $\rho_{uv}^{i}$ is the Spearman’s rank correlation coefficient between connectivity patterns of node *i* for subjects *u* and *v*. The number of altered regions when comparing pairs of groups using this non-parametric distance definition is depicted in Table 6. Apart from small changes, the scenario found using Pearson correlation is substantially confirmed: converting MCI have the maximal altered pattern w.r.t. healthy conditions. Moreover, the relevant regions showed in Figs 1 and 4 are also found to be significantly discriminating using the non-parametric distance.

**Table 6:** Number of significantly altered pattern connectivities of regions between groups after false discovery rate correction at 1% and 5% (in parenthesis) using Spearman correlation between connectivity patterns.

	HC	ncMCI	cMCI	AD
HC	-	3 (26)	284 (413)	8 (327)
ncMCI	3 (26)	-	0 (0)	4 (17)
cMCI	284 (413)	0 (0)	-	5 (7)
AD	8 (327)	4 (17)	5 (7)	-

## Discussion

The aim of this paper was to analyze MR images of MCI and AD patients in order to characterize their altered structural connectivity pattern compared to healthy subjects. Individual structural MRI networks were evaluated as the Pearson correlation of the intensity of voxels in the patches corresponding to nodes. Then, we performed the MDMR analysis to each pair of groups, so as to identify brain regions with different connectivity pattern in the two groups.

We remark that here we were not interested in analyzing topological metrics of brain networks: in the structural MRI newtorks, these properties have been already studied, pointing out, e.g., that AD networks have abnormal small world property w.r.t. networks of healthy subjects [44–46]. In [28] the path length, the clustering coefficient and the betweenness centrality were studied in controls and AD patients: altered path length of the parahippocampal gyrus, hippocampus, fusiform gyrus and precuneous showed a strong relationship with cognitive decline. Moreover, we stress that the definition of connectivity here used corresponds just to image similarity between patches and does not have an immediate neurophysiological interpretation, indeed two patches can be similar also in the absence of axonal connectivity.

The first interesting result here was the observation that MCI subjects converting to AD have the maximal altered pattern w.r.t. healthy conditions (the minimal average strength in the four groups), indeed ncMCI show only four significant regions; AD subjects thus show a partial recovery of connectivity, in the sense that many regions, that were altered in cMCI group, are not recognized altered in AD. The apparently paradoxical finding that in AD the structural networks are less altered than in cMCI lies in the fact that in AD there is more abundance of atrophic tissues. Consider two patches from an healthy brain and the corresponding connectivity (as measured by the Pearson correlation of intensities); now let us assume that one out of the two patches is affected by atrophy, then we expect a relevant alteration of their connectivity w.r.t. healthy conditions. On the other hand, when also the second patch becomes affected by atrophy, it is likely that the connectivity of the two patches gets back closer to the value of healthy brains. Moreover it is worth to mention that it has been found that the mean atrophy rates of ncMCI subjects are more similar to controls, whereas the mean atrophy rate of cMCI are more similar to AD [47]: this explains why we found a much lower degree of alterations in ncMCI than in cMCI. Also the analysis of the mean strength of the brain networks confirmed this scenario.

Furthermore, five regions were altered in AD but not in MCI conditions, i.e. their disconnection appears only when the AD disease sets in. The five regions, are depicted in Fig (4) and correspond to interesting anatomical structures such as hippocampus, whose altered neurogenesis has been shown to represent an early critical event in the course of AD [48], amygdala and cingulate gyrus [49], and others.

Secondly, comparing AD with ncMCI and cMCI, we found few significant nodes, in particular we identified six nodes with the following properties: (i) they are significant in the comparisons ncMCI-AD and cMCI-AD (ii) they contain all the four significant regions of both comparisons HC-ncMCI and HD-cMCI (iii) they are not identified as altered in the comparison HC-AD. These nodes form a connected brain region (which correspond to parahippocampal gyrus [50] subcallosal cortex [51] and other structures already studied in existing literatures [52]) with reduced strength of connectivity in the MCI stages; its structural connectivity, in AD, was statistically indistinguishable from those of healthy subjects. Alteration of this regions is thus characteristic of the MCI condition. This peculiar behavior is clearly shown by Fig (3).

Remarkably, two brain regions were significant only for ncMCI and correspond to hippocampus, amygdala, planum polare and subcallosal cortex.

Finally, the comparison of ncMCI and cMCI did not identify any significant region, but projecting the connectivity maps of MCI subjects onto the average connectome of healthy subjects lead to identify three brain regions that are significantly more altered in cMCI than in ncMCI. All these three regions correspond to the Frontal Orbital Cortex, which contains the secondary taste cortex, in which the reward value of taste is represented. It is also involved in emotional enhancement of memory [53]. The damage of the orbitofrontal cortex in AD is well known [54].

It is worth mentioning that all the five patches, which were identified as relevant for the ncMCI-cMCI comparison, belong to the left hemisphere, in accordance with previous results [55]. The structural connectivity pattern of these regions can thus provide useful information for predicting AD disease and, together with other indicators, such as Voxel Based Morphometry [56], A*β* brain networks [57], functional and DTI networks, can contribute to the construction of more efficient machine learning tools tailored to this important task.

Notably, we have also showed that by using a different distance measure for connectivity patterns, even though the number of significant nodes slightly changes, the overall scenario established by nodes depicted in Figs 1 and 4 remains consistent, thus assessing the robustness of MDMR method in concordance with [37]. Likewise, our method also suffers from the limitations noted in [37], in particular we would like to stress the sensitivity loss of highly focused areas related with group difference due to the fact of taking whole-brain pattern connectivity. In our case, this issue might be relevant in comparisons with MCI stages where the onset of cognitive deterioration takes place in small regions and then spread to the rest of the brain. As a consequence, an analysis within specific regions rather than the full brain should be performed to address this issue.

In addition, it is also important to clarify that regions found significant do not directly lead to high accuracy when used as features to classify between group labels [58]. Rather, our method seeks for changes in brain pattern connectivity that correlate significantly with a phenotypic variable, *i.e.* group label, within an exploratory analysis. Recent classification applications using MRI data that optimise prediction in AD/MCI diagnosis can be found in [59–61].

## Conclusions

Summarizing, our results confirm that individual structural MRI networks can be used to measure the departure from the healthy pattern, in MCI and AD patients. The definition of connectivity, here used, depends only on the image similarity of the intensity patterns in patches: it follows that alterations in the connectivity are due to atrophy and that these networks provide a geometrical representation of the progression of atrophy in AD. Since the connectivity of two atrophic patches can be similar to those of two healthy patches, the most altered connectivity is observed in the intermediate cMCI stage. Individual MRI-based networks are thus promising brain network which bring complementary information w.r.t. individual fMRI and DTI brain networks for the study of neurodegenerative diseases, and MDMR is a valuable method to study alterations of the connectivity pattern.

## References

[1] Wimo A, Ljunggren G, Winblad B. Costs of dementia and dementia care: a review. International Journal of Geriatric Psychiatry. 1997;12: 841–856. doi: 10.1002/(SICI)1099-1166(199708)12:8%3C841::AID-GPS652%3E3.0.CO;2-R 9283930

[2] Hardy J. Alzheimer’s disease: the amyloid cascade hypothesis: an update and reappraisal. J Alzheimers Dis. 2006;9: 151–153. doi: 10.3233/JAD-2006-9S317 16914853 10.3233/jad-2006-9s317

[3] Mueller SG, Weiner MW, Thal LJ, Petersen RC, Jack C, Jagust W, et al The Alzheimer’s Disease Neuroimaging Initiative. Neuroimaging Clin N Am. 2005;15: 869–xii. doi: 10.1016/j.nic.2005.09.008 16443497 10.1016/j.nic.2005.09.008 PMC2376747

[4] Cheng B, Liu M, Suk H-I, Shen D, Zhang D, Alzheimer’s Disease Neuroimaging Initiative. Multimodal manifold-regularized transfer learning for MCI conversion prediction. Brain Imaging Behav. 2015;9: 913–926. doi: 10.1007/s11682-015-9356-x 25702248 10.1007/s11682-015-9356-x PMC4546576

[5] Li H, Liu Y, Gong P, Zhang C, Ye J. Hierarchical Interactions Model for Predicting Mild Cognitive Impairment (MCI) to Alzheimer’s Disease (AD) Conversion. PLoS One. 2014;9: e82450 doi: 10.1371/journal.pone.0082450 24416143 10.1371/journal.pone.0082450 PMC3885394

[6] Ritter K, Schumacher J, Weygandt M, Buchert R, Allefeld C, Haynes J-D. Multimodal prediction of conversion to Alzheimer’s disease based on incomplete biomarkers. Alzheimer’s & Dementia: Diagnosis, Assessment & Disease Monitoring. 2015;1: 206–215. 10.1016/j.dadm.2015.01.006 PMC4877756 27239505

[7] Mitchell AJ, Shiri-Feshki M. Rate of progression of mild cognitive impairment to dementia—meta-analysis of 41 robust inception cohort studies. Acta Psychiatrica Scandinavica. 2009;119: 252–265. doi: 10.1111/j.1600-0447.2008.01326.x 19236314 10.1111/j.1600-0447.2008.01326.x

[8] Petersen RC, Roberts RO, Knopman DS, Geda YE, Cha RH, Pankratz VS, et al Prevalence of mild cognitive impairment is higher in men. The Mayo Clinic Study of Aging. Neurology. 2010;75: 889–897. doi: 10.1212/WNL.0b013e3181f11d85 20820000 10.1212/WNL.0b013e3181f11d85 PMC2938972

[9] Schmidtke K, Hermeneit S. High rate of conversion to Alzheimer’s disease in a cohort of amnestic MCI patients. Int Psychogeriatr. 2008;20: 96–108. doi: 10.1017/S1041610207005509 17506911 10.1017/S1041610207005509

[10] Rozzini L, Chilovi BV, Conti M, Bertoletti E, Delrio I, Trabucchi M, et al Conversion of amnestic Mild Cognitive Impairment to dementia of Alzheimer type is independent to memory deterioration. Int J Geriatr Psychiatry. 2007;22: 1217–1222. doi: 10.1002/gps.1816 17562522 10.1002/gps.1816

[11] Geslani DM, Tierney MC, Herrmann N, Szalai JP. Mild cognitive impairment: an operational definition and its conversion rate to Alzheimer’s disease. Dement Geriatr Cogn Disord. 2005;19: 383–389. doi: 10.1159/000084709 15802914 10.1159/000084709

[12] Zhang S, Smailagic N, Hyde C, Noel-Storr AH, Takwoingi Y, McShane R, et al ^11^C-PIB-PET for the early diagnosis of Alzheimer’s disease dementia and other dementias in people with mild cognitive impairment (MCI) In:The Cochrane Collaboration, editor. Cochrane Database of Systematic Reviews. Chichester, UK:John Wiley & Sons, Ltd; 2014. 10.1002/14651858.CD010386.pub2 PMC6464750 25052054

[13] Teipel S, Drzezga A, Grothe MJ, Barthel H, Chételat G, Schuff N, et al Multimodal imaging in Alzheimer’s disease: validity and usefulness for early detection. Lancet Neurol. 2015;14: 1037–1053. doi: 10.1016/S1474-4422(15)00093-9 26318837 10.1016/S1474-4422(15)00093-9

[14] Bellotti R, Pascazio S. Editorial: Advanced physical methods in brain research. Eur Phys J Plus. 127: 145 doi: 10.1140/epjp/i2012-12145-4

[15] Westman E, Cavallin L, Muehlboeck J-S, Zhang Y, Mecocci P, Vellas B, et al Sensitivity and specificity of medial temporal lobe visual ratings and multivariate regional MRI classification in Alzheimer’s disease. PLoS ONE. 2011;6: e22506 doi: 10.1371/journal.pone.0022506 21811624 10.1371/journal.pone.0022506 PMC3141068

[16] Eskildsen SF, Coupé P, García-Lorenzo D, Fonov V, Pruessner JC, Collins DL, et al Prediction of Alzheimer’s disease in subjects with mild cognitive impairment from the ADNI cohort using patterns of cortical thinning. Neuroimage. 2013;65: 511–521. doi: 10.1016/j.neuroimage.2012.09.058 23036450 10.1016/j.neuroimage.2012.09.058 PMC4237400

[17] Liu Y, Mattila J, Ruiz MÁM, Paajanen T, Koikkalainen J, van Gils M, et al Predicting AD conversion: comparison between prodromal AD guidelines and computer assisted PredictAD tool. PLoS ONE. 2013;8: e55246 doi: 10.1371/journal.pone.0055246 23424625 10.1371/journal.pone.0055246 PMC3570420

[18] Sporns O, Tononi G, Kötter R. The Human Connectome: A Structural Description of the Human Brain. PLoS Comput Biol. 2005;1: e42 doi: 10.1371/journal.pcbi.0010042 16201007 10.1371/journal.pcbi.0010042 PMC1239902

[19] Biswal BB, Mennes M, Zuo X-N, Gohel S, Kelly C, Smith SM, et al Toward discovery science of human brain function. Proc Natl Acad Sci USA. 2010;107: 4734–4739. doi: 10.1073/pnas.0911855107 20176931 10.1073/pnas.0911855107 PMC2842060

[20] Biswal B, Yetkin FZ, Haughton VM, Hyde JS. Functional connectivity in the motor cortex of resting human brain using echo-planar MRI. Magn Reson Med. 1995;34: 537–541. doi: 10.1002/mrm.1910340409 8524021 10.1002/mrm.1910340409

[21] Hagmann P, Cammoun L, Gigandet X, Meuli R, Honey CJ, Wedeen VJ, et al Mapping the structural core of human cerebral cortex. PLoS Biol. 2008;6: e159 doi: 10.1371/journal.pbio.0060159 18597554 10.1371/journal.pbio.0060159 PMC2443193

[22] He Y, Chen ZJ, Evans AC. Small-world anatomical networks in the human brain revealed by cortical thickness from MRI. Cereb Cortex. 2007;17: 2407–2419. doi: 10.1093/cercor/bhl149 17204824 10.1093/cercor/bhl149

[23] Bassett DS, Bullmore E, Verchinski BA, Mattay VS, Weinberger DR, Meyer-Lindenberg A. Hierarchical organization of human cortical networks in health and schizophrenia. J Neurosci. 2008;28: 9239–9248. doi: 10.1523/JNEUROSCI.1929-08.2008 18784304 10.1523/JNEUROSCI.1929-08.2008 PMC2878961

[24] Alexander-Bloch A, Giedd JN, Bullmore E. Imaging structural co-variance between human brain regions. Nat Rev Neurosci. 2013;14: 322–336. doi: 10.1038/nrn3465 23531697 10.1038/nrn3465 PMC4043276

[25] Tijms BM, Seriès P, Willshaw DJ, Lawrie SM. Similarity-based extraction of individual networks from gray matter MRI scans. Cereb Cortex. 2012;22: 1530–1541. doi: 10.1093/cercor/bhr221 21878484 10.1093/cercor/bhr221

[26] Wang H, Jin X, Zhang Y, Wang J. Single-subject morphological brain networks: connectivity mapping, topological characterization and test-retest reliability. Brain Behav. 2016;6: e00448 doi: 10.1002/brb3.448 27088054 10.1002/brb3.448 PMC4782249

[27] La Rocca M. et al A multiplex network model to characterize brain atrophy in structural MRI In:Mantica G, Stoop R, Stramaglia S, editors. Emergent Complexity from Nonlinearity, in Physics, Engineering and the Life Sciences. Springer Proceedings in Physics 191; 2017 pp 189–198.

[28] Tijms BM, Möller C, Vrenken H, Wink AM, de Haan W, van der Flier WM, et al Single-Subject Grey Matter Graphs in Alzheimer’s Disease. PLoS One. 2013;8 doi: 10.1371/journal.pone.0058921 23536835 10.1371/journal.pone.0058921 PMC3594199

[29] Tijms BM, Yeung HM, Sikkes SAM, Möller C, Smits LL, Stam CJ, et al Single-Subject Gray Matter Graph Properties and Their Relationship with Cognitive Impairment in Early- and Late-Onset Alzheimer’s Disease. Brain Connectivity. 2014;4: 337–346. doi: 10.1089/brain.2013.0209 24735020 10.1089/brain.2013.0209

[30] Tijms BM, Sprooten E, Job D, Johnstone EC, Owens DGC, Willshaw D, et al Grey matter networks in people at increased familial risk for schizophrenia. Schizophrenia Research. 2015;168: 1–8. doi: 10.1016/j.schres.2015.08.025 26330380 10.1016/j.schres.2015.08.025

[31] Bullmore ET, Bassett DS. Brain graphs: graphical models of the human brain connectome. Annu Rev Clin Psychol. 2011;7: 113–140. doi: 10.1146/annurev-clinpsy-040510-143934 21128784 10.1146/annurev-clinpsy-040510-143934

[32] Çiftçi K. Minimum spanning tree reflects the alterations of the default mode network during Alzheimer’s disease. Ann Biomed Eng. 2011;39: 1493–1504. doi: 10.1007/s10439-011-0258-9 21286814 10.1007/s10439-011-0258-9

[33] Sporns O. Network attributes for segregation and integration in the human brain. Curr Opin Neurobiol. 2013;23: 162–171. doi: 10.1016/j.conb.2012.11.015 23294553 10.1016/j.conb.2012.11.015

[34] Suk H-I, Lee S-W, Shen D. Hierarchical Feature Representation and Multimodal Fusion with Deep Learning for AD/MCI Diagnosis. Neuroimage. 2014;101: 569–582. doi: 10.1016/j.neuroimage.2014.06.077 25042445 10.1016/j.neuroimage.2014.06.077 PMC4165842

[35] Davatzikos C. Why voxel-based morphometric analysis should be used with great caution when characterizing group differences. Neuroimage. 2004;23: 17–20. doi: 10.1016/j.neuroimage.2004.05.010 15325347 10.1016/j.neuroimage.2004.05.010

[36] Amoroso N, Errico R, Bruno S, Chincarini A, Garuccio E, Sensi F, et al Hippocampal unified multi-atlas network (HUMAN): protocol and scale validation of a novel segmentation tool. Phys Med Biol. 2015;60: 8851–8867. doi: 10.1088/0031-9155/60/22/8851 26531765 10.1088/0031-9155/60/22/8851

[37] Shehzad Z, Kelly C, Reiss PT, Cameron Craddock R, Emerson JW, McMahon K, et al A multivariate distance-based analytic framework for connectome-wide association studies. Neuroimage. 2014;93 Pt 1:74–94. doi: 10.1016/j.neuroimage.2014.02.024 24583255 10.1016/j.neuroimage.2014.02.024 PMC4138049

[38] Wee C-Y, Yap P-T, Shen D, Alzheimer’s Disease Neuroimaging Initiative. Prediction of Alzheimer’s disease and mild cognitive impairment using cortical morphological patterns. Hum Brain Mapp. 2013;34: 3411–3425. doi: 10.1002/hbm.22156 22927119 10.1002/hbm.22156 PMC3511623

[39] Boccardi M, Bocchetta M, Morency FC, Collins DL, Nishikawa M, Ganzola R, et al Training labels for hippocampal segmentation based on the EADC-ADNI harmonized hippocampal protocol. Alzheimers Dement. 2015;11: 175–183. doi: 10.1016/j.jalz.2014.12.002 25616957 10.1016/j.jalz.2014.12.002

[40] Jenkinson M, Beckmann CF, Behrens TEJ, Woolrich MW, Smith SM. FSL. Neuroimage. 2012;62: 782–790. doi: 10.1016/j.neuroimage.2011.09.015 21979382 10.1016/j.neuroimage.2011.09.015

[41] Desikan RS, Ségonne F, Fischl B, Quinn BT, Dickerson BC, Blacker D, et al An automated labeling system for subdividing the human cerebral cortex on MRI scans into gyral based regions of interest. Neuroimage. 2006;31: 968–980. doi: 10.1016/j.neuroimage.2006.01.021 16530430 10.1016/j.neuroimage.2006.01.021

[42] Daniel BM. MDMR: Multivariate Distance Matrix Regression. R package version 0.4.0. 2016.

[43] Kruskal JB. Multidimensional scaling by optimizing goodness of fit to a nonmetric hypothesis. Psychometrika. 1964;29: 1–27. doi: 10.1007/BF02289565

[44] He Y, Chen Z, Evans A. Structural insights into aberrant topological patterns of large-scale cortical networks in Alzheimer’s disease. J Neurosci. 2008;28: 4756–4766. doi: 10.1523/JNEUROSCI.0141-08.2008 18448652 10.1523/JNEUROSCI.0141-08.2008 PMC6670444

[45] Yao Z, Zhang Y, Lin L, Zhou Y, Xu C, Jiang T, et al Abnormal cortical networks in mild cognitive impairment and Alzheimer’s disease. PLoS Comput Biol. 2010;6: e1001006 doi: 10.1371/journal.pcbi.1001006 21124954 10.1371/journal.pcbi.1001006 PMC2987916

[46] Desikan RS, Sabuncu MR, Schmansky NJ, Reuter M, Cabral HJ, Hess CP, et al Selective disruption of the cerebral neocortex in Alzheimer’s disease. PLoS ONE. 2010;5: e12853 doi: 10.1371/journal.pone.0012853 20886094 10.1371/journal.pone.0012853 PMC2944799

[47] Leung KK, Bartlett JW, Barnes J, Manning EN, Ourselin S, Fox NC. Cerebral atrophy in mild cognitive impairment and Alzheimer disease. Neurology. 2013;80: 648–654. doi: 10.1212/WNL.0b013e318281ccd3 23303849 10.1212/WNL.0b013e318281ccd3 PMC3590059

[48] Mu Y, Gage FH. Adult hippocampal neurogenesis and its role in Alzheimer’s disease. Mol Neurodegener. 2011;6: 85 doi: 10.1186/1750-1326-6-85 22192775 10.1186/1750-1326-6-85 PMC3261815

[49] Dinomais M, Celle S, Duval GT, Roche F, Henni S, Bartha R, et al Anatomic Correlation of the Mini-Mental State Examination: A Voxel-Based Morphometric Study in Older Adults. PLoS ONE. 2016;11: e0162889 doi: 10.1371/journal.pone.0162889 27741236 10.1371/journal.pone.0162889 PMC5065203

[50] Mattis PJ, Niethammer M, Sako W, Tang CC, Nazem A, Gordon ML, et al Distinct brain networks underlie cognitive dysfunction in Parkinson and Alzheimer diseases. Neurology. 2016;87: 1925–1933. doi: 10.1212/WNL.0000000000003285 27708130 10.1212/WNL.0000000000003285 PMC5100716

[51] Lindberg O, Westman E, Karlsson S, Östberg P, Svensson LA, Simmons A, et al Is the subcallosal medial prefrontal cortex a common site of atrophy in Alzheimer’s disease and frontotemporal lobar degeneration? Front Aging Neurosci. 2012;4: 32 doi: 10.3389/fnagi.2012.00032 23189052 10.3389/fnagi.2012.00032 PMC3504956

[52] Zhang Y, Dong Z, Phillips P, Wang S, Ji G, Yang J, et al Detection of subjects and brain regions related to Alzheimer’s disease using 3D MRI scans based on eigenbrain and machine learning. Front Comput Neurosci. 2015;9: 66 doi: 10.3389/fncom.2015.00066 26082713 10.3389/fncom.2015.00066 PMC4451357

[53] Kumfor F, Irish M, Hodges JR, Piguet O. The orbitofrontal cortex is involved in emotional enhancement of memory: evidence from the dementias. Brain. 2013;136: 2992–3003. doi: 10.1093/brain/awt185 23838694 10.1093/brain/awt185

[54] Van Hoesen GW, Parvizi J, Chu CC. Orbitofrontal cortex pathology in Alzheimer’s disease. Cereb Cortex. 2000;10: 243–251. doi: 10.1093/cercor/10.3.243 10731219 10.1093/cercor/10.3.243

[55] Derflinger S, Sorg C, Gaser C, Myers N, Arsic M, Kurz A, et al Grey-matter atrophy in Alzheimer’s disease is asymmetric but not lateralized. J Alzheimers Dis. 2011;25: 347–357. 21422522 10.3233/JAD-2011-110041

[56] Ashburner J, Friston KJ. Voxel-based morphometry—the methods. Neuroimage. 2000;11: 805–821. doi: 10.1006/nimg.2000.0582 10860804 10.1006/nimg.2000.0582

[57] Duan H, Jiang J, Xu J, Zhou H, Huang Z, Yu Z, et al Differences in A*β* brain networks in Alzheimer’s disease and healthy controls. Brain Research. 2017;1655: 77–89. doi: 10.1016/j.brainres.2016.11.019 27867033 10.1016/j.brainres.2016.11.019

[58] Lo A, Chernoff H, Zheng T, Lo S-H. Why significant variables aren’t automatically good predictors. Proc Natl Acad Sci U S A. 2015;112: 13892–13897. doi: 10.1073/pnas.1518285112 26504198 10.1073/pnas.1518285112 PMC4653162

[59] Liu M, Zhang D, Shen D. Relationship Induced Multi-Template Learning for Diagnosis of Alzheimer’s Disease and Mild Cognitive Impairment. IEEE Trans Med Imaging. 2016;35: 1463–1474. doi: 10.1109/TMI.2016.2515021 26742127 10.1109/TMI.2016.2515021 PMC5572669

[60] Liu M, Zhang D, Shen D, the Alzheimer’s Disease Neuroimaging Initiative. View-centralized multi-atlas classification for Alzheimer’s disease diagnosis: View-Centralized Multi-Atlas Classification. Human Brain Mapping. 2015;36: 1847–1865. 25624081 10.1002/hbm.22741 PMC6869465

[61] Liu M, Zhang D, Adeli-Mosabbeb E, Shen D. Inherent Structure Based Multi-view Learning with Multi-template Feature Representation for Alzheimer’s Disease Diagnosis. IEEE Trans Biomed Eng. 2016;63: 1473–1482. doi: 10.1109/TBME.2015.2496233 26540666 10.1109/TBME.2015.2496233 PMC4851920

